# Microarray Gene Expression Analysis of Tumorigenesis and Regional Lymph Node Metastasis in Laryngeal Squamous Cell Carcinoma

**DOI:** 10.1371/journal.pone.0084854

**Published:** 2013-12-26

**Authors:** Meng Lian, Jugao Fang, Demin Han, Hongzhi Ma, Ling Feng, Ru Wang, Fan Yang

**Affiliations:** 1 Department of Otorhinolaryngology Head and Neck Surgery, Beijing Tongren Hospital, Capital Medical University, Beijing, China; 2 Key Laboratory of Otorhinolaryngology Head and Neck Surgery, Ministry of Education, Beijing Institute of Otorhinolaryngology, Beijing, China; West Virginia University, United States of America

## Abstract

**Background:**

Laryngeal squamous cell carcinoma (LSCC) is the most common type in head and neck squamous cell carcinoma (HNSCC), and the development and progression of LSCC are multistep processes accompanied by changes of molecular biology.

**Objective:**

The purpose of this study was to investigate the molecular basis of tumorigenesis and regional lymph node metastasis in LSCC, and provide a set of genes that may be useful for the development of novel diagnostic markers and/or more effective therapeutic strategies.

**Methods:**

A total number of 10 patients who underwent surgery for primary laryngeal squamous cell carcinoma were recruited for microarray analysis. LSCC tissues compared with corresponding adjacent non-neoplastic tissues were analysed by Illumina mRNA microarrays, and LSCC tissues with regional lymph node metastasis and LSCC tissues without regional lymph node metastasis were analyzed in the same manner. The most frequently differently expressed genes screened by microarrays were also validated by qRT-PCR in another 42 patients diagnosed for LSCC.

**Results:**

Analysed by Illumina mRNA microarrays, there were 361 genes significantly related to tumorigenesis while 246 genes significantly related to regional lymph node metastasis in LSCC. We found that the six genes (CDK1, CDK2, CDK4, MCM2, MCM3, MCM4) were most frequently differently expressed functional genes related to tumorigenesis while eIF3a and RPN2 were most frequently differently expressed functional genes related to regional lymph node metastasis in LSCC. The expressions of these genes were also validated by qRT-PCR.

**Conclusions:**

The research revealed a gene expression signature of tumorigenesis and regional lymph node metastasis in laryngeal squamous cell carcinoma. Of the total, the deregulation of several genes (CDK1, CDK2, CDK4, MCM2, MCM3, MCM4, EIF3a and RPN2) were potentially associated with disease development and progression. The result will contribute to the understanding of the molecular basis of LSCC and help to improve diagnosis and treatment.

## Introduction

Head and neck squamous cell carcinoma (HNSCC) is the sixth most frequent cancer, and laryngeal squamous cell carcinoma (LSCC) is the most common type, accounting for 1% to 2% of all malignancies worldwide [1-3]. Corresponding to ~25% of HNSCC cases, of which the long-term survival rate has remained at 50%, LSCC should be drawn attention [4]. However, cervical lymph nodes metastases and distant metastases are the factors that significantly affect the prognosis in LSCC patients [5]. An important step in the process of tumor metastases is the detachment of malignant cells from their original site [6]. In normal epithelial tissues, cell–cell adhesion is mediated by a large number of cell adhesion molecules. Defective interactions between adhesion molecules play a critical role in oncogenesis and metastasis [7]. Metastasis to a regional lymph node is the first indication of tumor metastasis competence [8].The understanding of the process involving this lymphatic progression will help to handle the treatment of these aggressive tumors. Neoplastic cells with metastasis capacity acquire distinct cellular capabilities, such as the ability to proliferate without limit, to evade apoptosis, to escape immune surveillance, and to express factors that alter the growth of blood and lymphatic vessels so as to create conduits for tumor metastasis [9].

 The development and progression of LSCC are multistep processes accompanied by changes of molecular biology. Various studies have revealed numerous molecular abnormalities in LSCC, including MMP-2 [10], HER2 [11], E-cadherin [12], AEG-1 [13], EGFL7 [14], NF-kappaB [15], CXCR2 [16] and so on. However, the complete array of molecular changes that occur during oncogenesis and metastasis remains elusive. 

 Genome-wide analysis using microarrays has emerged as an important tool for biological studies by allowing the large-scale identification and comprehensive analysis of gene expression profiles and mutation mapping. This technology has enabled discovery of large gene-sets whose altered expression levels reflect specific disease status or given treatment response, which provides clues of related biological mechanisms. The data of this technology also could be recognized as an effective method for validation of biomarkers, discovery of gene functions, and development of new drugs targeting specific genes [17]. Large-scale studies involving microarrays have identified specific gene expression signatures associated with expression changes in HNSCC tissue samples compared to normal tissue [18]. However, the microarray gene expression studies on LSCC with regional lymph node metastasis need to be further explored.

 In the present study, we focus on the gene dysregulation about tumorigenesis and regional lymph node metastasis in LSCC. A number of ten laryngeal squamous cell carcinoma tissues and corresponding adjacent non-neoplastic tissues were recruited. We constructed an mRNA microarray platform containing probes for 34601 genes. Gene dysregulation related with tumorigenesis and regional lymph node metastasis was analyzed by biological analysis software, and significant and functional gene dysregulation was validated further. Our findings contribute to the understanding of the molecular basis of tumorigenesis and regional lymph node metastasis in LSCC, and provide a set of genes that may be useful for the development of novel diagnostic markers and/or more effective therapeutic strategies.

## Materials and Methods

All patients we had selected were treated in Department of Head and Neck Surgery, Beijing Tongren Hospital, and all patients provided written informed consent before their participation. The Ethics Committee of Capital Medical University approval was obtained for the use of all samples by using a protocol that conforms to the provisions of the Declaration of Helsinki.

### Tissue samples and patients

A total number of 10 patients (no females) who underwent surgery for primary laryngeal squamous cell carcinoma were recruited for microarray gene expression analysis, and the TNM stage of each patient was determined as Table **1**. A second group of 42 patients (no female**s**) who underwent surgery for primary LSCC were also recruited for qRT-PCR, and the TNM stage of each patient was determined as Table **2**. The two patient cohorts used for microarrays and qRT-PCR investigations were separated. The cancer tissues and corresponding adjacent non-neoplastic tissues were collected during surgery. Each specimen was immediately snap-frozen in liquid nitrogen and stored at -80 °C for subsequent study. The pathology of all the cancer tissues were squamous cell carcinoma, which were evaluated by pathologists. 

**Table 1:** Clinical data of patients for microarrays.

Tumor tissues	age	T	N	M	corresponding non-neoplastic tissues
WA	53	2	0	0	WB
WC	55	4	3	0	WD
WE	52	3	0	0	WF
WG	56	4	0	0	WH
WI	74	3	0	0	WJ
WK	64	1	0	0	WL
LA	71	4	2a	0	LB
LC	58	4	2b	0	LD
LE	52	3	2b	0	LF
LG	66	3	2c	0	LH

**Table 2:** Clinical data of patients for qRT-PCR.

TNM	number	average age
T1N0M0	1	51
T2N0M0	6	47
T3N0M0	8	56
T4N0M0	8	60
T3NxM0(X≠0)	9	62
T4NxM0(X≠0)	10	59

### RNA extraction and quality assessment

Total RNA was extracted from tissue samples using Trizol Reagent (Invitrogen). Then the RNA quantity was determined using denaturing gel electrophoresis which produced at least 2 distinct bands representing the 28S and 18S ribosomal RNA, confirming that the total RNA was not contaminated with DNA and the RNA was not degraded.

### cRNA amplification

Reverse transcription to synthesize first strand cDNA was primed with the T7 Oligo(dT) Primer to synthesize cDNA containing a T7 promoter sequence.Second Strand cDNA Synthesis converted the single-stranded cDNA into a double-stranded DNA (dsDNA) template for transcription.The reaction employed DNA Polymerase and RNase H to simultaneously degrade the RNA and synthesize second strand cDNA. Then, cDNA purification removed RNA, primers, enzymes, and salts that would inhibit in vitro transcription.After that, in vitro transcription to synthesize cRNA generated multiple copies of biotinylated cRNA from the double-stranded cDNA templates.At last, cRNA purification removed unincorporated NTPs, salts, enzymes, and inorganic phosphate. After purification, the cRNA was ready for use with Illumina’s direct hybridization array kits.

### Illumina Human HT-12 BeadChip

The RNA samples which passed the quality test were hybridized with reagents for hybridization according to protocols.Hatched in room temperature,and then processed through high-temperature wash and ethanol wash.After three room- temperature washes, image could be read by the software called Illumina Bead Chip Reader. The dataset had been submitted to Gene Expression Omnibus, and the accession number was GSE51985.

### Quantitative real-time PCR

The transcriptional level of the target genes were measured by qRT-PCR detection.Trizol was applied to extract total cellular RNA. Prepared Template RNA (5μl) / primer (1μl) mixture in Microtube tube. Keep in 70 °C for 10 minutes, then rapid quenched in ice no more than 5 minutes. After that, centrifuged for a few seconds so that the template RNA / primer solution of denatured aggregation gathered at the bottom of the tube Microtube. Then added 5 × M-MLV Buffer, RNase Inhibitor and dNTP Mixture preparation called reverse transcription reaction solution in the Microtube tube, 4μl totally. This solution had to keep in 42 °C for 1 hour. Cooled by ice after hold in 95 °C for 15 minutes, then we got the cDNA solution. Mixed this 1μl cDNA solution, Taq DNA Polymerase, 2XSYBR to 20μl the mixed system. Hold it in 95 °C for 5 mins for denaturating, then followed by 45 cycles totally which were keeping in 95 °C for 30s, keeping in 65 °C for 30s, and keeping in 72 °C for 5 mins. The gene expression levels were determined based on Livak method [19].The results that 2^-ΔΔCT^ values of all samples were analysed automatically by computer control with β-actin gene as an internal reference. The primer sequences as Table **3**:

**Table 3:** Primer sequences.

Gene	Primer Sequence
β-actin	F:GTGAAGGTGACAGCAGTCGGTT
	R:AGTGGGGTGGCTTTTAGGA
CDK1	F:CTTTTATGTCTTGCTTAAGT
	R:CATAATTCTAAATAAAACTG
CDK2	F:CTCCTACCCCATAGGAGTTAG
	R:GTCCAATATAGGTAATCATC
CDK4	F:CTTTCCTGCAAAACCTTAAAG
	R:GGACTCCAGTCCTCAAGCTCTG
MCM2	F:CCTCTGTGCTTTATGGACAC
	R:GGAGGCTCACGAAACAGAGG
MCM3	F:GGTGATGAAGCTGAGTTCAGG
	R:CTGAAGACTCATGAAAACCC
MCM4	F:GTATTTTTTGGTAGAGACGGCTTC
	R:GTGACGTGGGTCGGAAAC
EIF3a	F:GTAAACATTACAAACATTGG
	R:GCGTTCACACTTAGGTTTGTC
RPN2	F:GGACTCAGCTCAACATGTTCCAG
	R:GATGCTTGTCGTGTAATCAAGG

### Microarray analysis of laryngeal squamous cell carcinoma tissues VS corresponding adjacent non-neoplastic tissues

Illumina Genomestudio-Gene Expression software was used for background correction and missing value difference processing,and then the data was normalized by quantitle method. Illumina Custom software was used for analyzing different gene expression.To reduce the false positive rate, SAM method was chosen for analyzing the different gene expression further by Mev software (FDR<0.05 was chosen). Paired t tests were used for analysis, and differences were considered statistically significant at P-value <0.05.

### Microarray analysis of LSCC tissues with regional lymph node metastasis VS cancer tissues without regional lymph node metastasis

Independent sample t tests were used for analysis, and other studies were conducted in the same way as the above.

### Pathway analysis

Recently, pathway-based methods have been developed to be adopted in the development of a disease or some other physiological process. Pathway-based methods are powerful tools that can give new insights into various biological phenomena from the system or functional levels [20]. The GO database provides a controlled vocabulary of terms to define biological descriptors (GO categories) and to support biologically meaningful annotation of gene products [21], while the KEGG pathways database has been widely used for the systematic analysis of gene functions that involve networks of molecular interactions in cells [22].

In this study, GO categories and KEGG pathways were used to identify pathways with considerable enrichment of the genes by on-line analysis called “Web-Based Gene Set Analysis Toolkit”. P-value was calculated using hypergeometric distribution and the cut-off was set at < 0.05.

### Statistical analysis for qRT-PCR

All data were imported to SPSS 20.0.The data which did not meet normality would be conversed into normality. Independent sample t tests or paired sample t tests were used for analysis involving two samples. Differences were considered statistically significant at P-value <0.05.

## Results

### Gene expression analysis of tumorigenesis in LSCC

**Figure 1:**
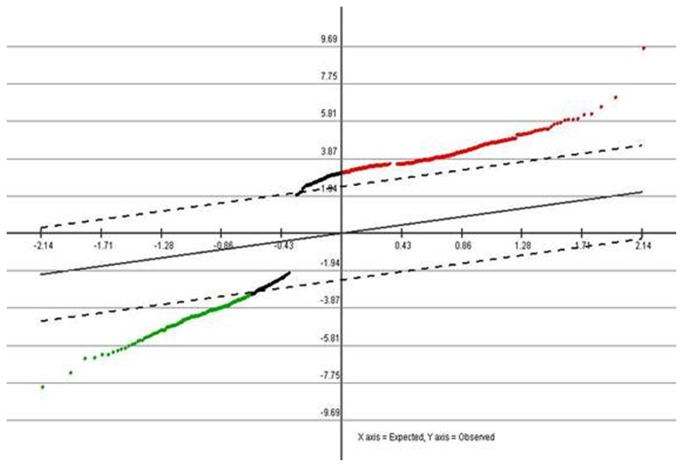
SAM Graph of genes related to tumorigenesis in LSCC. Red points indicated higher expression genes(P<0.05) and green points indicated lower expression genes(P<0.05) across the 361 samples.

**Figure 2:**
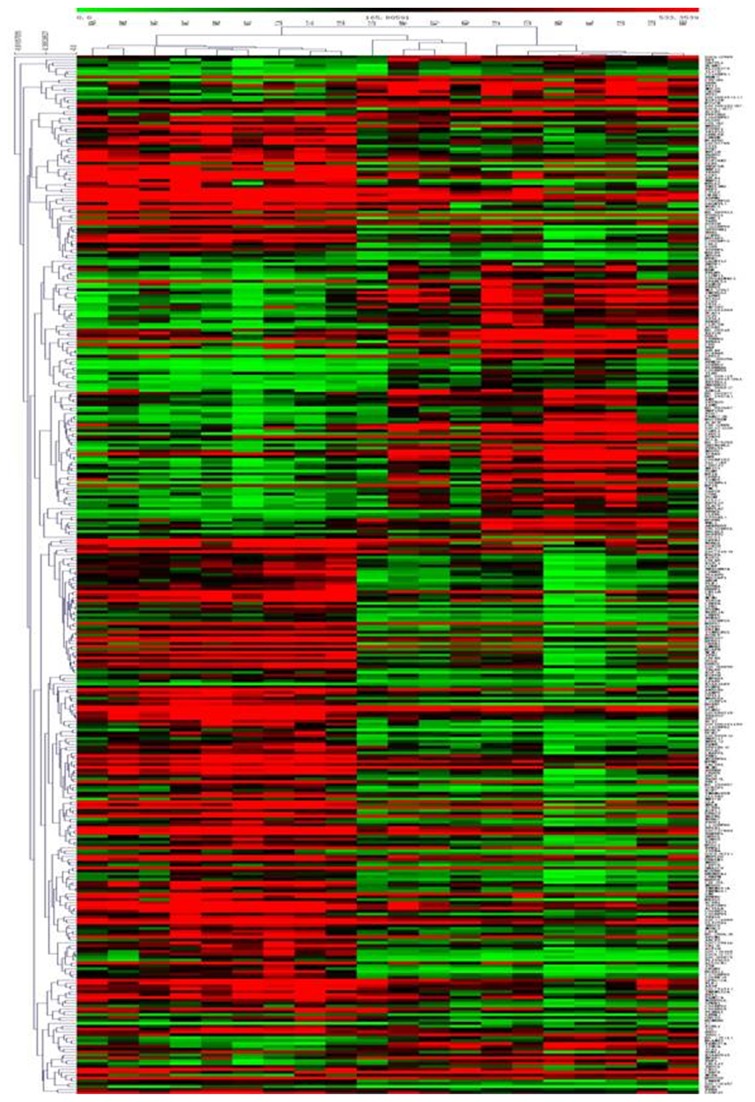
Hierarchical Trees: All significant genes of tumorigenesis in LSCC. For each gene (row), red indicated a higher expression and green a lower one relative to the average level of expression of that gene across the 361 samples (columns).

Expression analysis using the mRNA microarrays was initially performed on 10 laryngeal squamous cell carcinoma tissues and their corresponding adjacent non-neoplastic tissues. Of the 34601 genes analyzed, 361 genes showed statistically significant differences in the expression between LSCC tissues and corresponding non-neoplastic tissues (P <0.05). Among these 361genes, 232 showed a higher expression in tumor than in non-tumor tissue, and 129 presented the contrasting pattern. Supervised hierarchical clustering analysis revealed that the expression patterns of the selected set of 361 differentially expressed genes were able to perfectly distinguish tumors from non-neoplastic tissues in the set of samples, which suggested heterogeneity between cancer cases and normal tissues. The result was shown below in Figure 1 and Figure 2.

 To identify typical nuclear genes which might be diagnostic or therapeutic markers from the identified 361 genes, the GO database and the KEGG pathways database were used for biological process enrichment analysis. Analyzed by GO database,we observed that these genes were involved in processes such as mitosis, cell cycle phase, cell cycle process, ATP-banding, apoptosis, nuclear division and so on (P <0.05). DNA replication pathway, cell cycle pathway and p53 signaling pathway played especially important role in tumorigenesis of LSCC analyzed by KEGG pathways database(P<0.05).Of the 361 genes, we found that the six genes(CDK1, CDK2, CDK4, MCM2, MCM3, MCM4) were related with both cell cycle in GO database and DNA replication, cell cycle pathways in KEGG database. The six genes were overexpression in cancer tissues compared to adjacent non-neoplastic tissues, suggesting they might be useful target markers. The results were shown below in Table 4, Table 5 and Table 6.

**Table 4:** Microarray analysis of six genes between LSCC tissues and their corresponding adjacent non-neoplastic tissues.

genes	fold change	P
mcm2	3.58	0.005
mcm3	2.13	0.020
mcm4	2.74	0.008
CDK1	2.58	0.028
CDK2	2.15	0.025
CDK4	2.38	0.002

**Table 5:** Six genes in GO database ID.

GO database (ID)	genes	P
**GO:0000278**	CDK1,CDK2,CDK4,mcm2,mcm3,mcm4	6.68e-19
**GO:0022403**	CDK1,CDK2,CDK4,mcm2,mcm3,mcm4	1.66e-18
**GO:0022402**	CDK1,CDK2,CDK4,mcm2,mcm3,mcm4	3.75e-18
**GO:0007049**	CDK1,CDK2,CDK4,mcm2,mcm3,mcm4	2.13e-17
**GO:0007067**	CDK1,CDK2	2.71e-14
**GO:0000280**	CDK1,CDK2	2.71e-14
**GO:0000087**	CDK1,CDK2	4.75e-14
**GO:0000279**	CDK1,CDK2	3.00e-14

**Table 6:** Six genes in KEGG pathways database ID.

KEGG pathways database (ID)	genes	P
**Cell cycle(04110)**	CDK1,CDK2,CDK4,mcm2,mcm3,mcm4	0.0005
**p53 signaling pathway(04115)**	CDK1,CDK2,CDK4	0.00078
**DNA replication(03030**	mcm2,mcm3,mcm4	5.25e-07

 Drug association analysis database is one of pathway-based methods in “Web-Based Gene Set Analysis Toolkit”. The analysis of the 361 genes by this database as previously introduced suggested that CDK1 was related to paclitaxel, mechlorethamine and CDK2 was also related to mechlorethamine, which indicated CDK1 and CDK2 also might be therapeutic target genes. The results of the 2 genes in the pathway analysis ID (CDK1, CDK2) were shown below in Table 7.

**Table 7:** CDK1,CDK2 in drug association analysis database ID.

Drug association analysis database (ID)	genes	P
**Mechlorethamine (PA450336)**	CDK1,CDK2	0.0252
**Paclitaxel (PA450761)**	CDK1	0.0003

 Minichromosome maintenance proteins are essential for DNA replication in all eukaryotic cells and for restricting replication to once per cell cycle [23],and cyclin-dependent kinases (CDKs) interact at specific stages of the cell cycle to drive the cell cycle from one phase to the next [24]. To investigate whether the typical nuclear genes were able to distinguish LSCC from non-tumor larynx tissues, these 6 genes (CDK1, CDK2, CDK4, MCM2, MCM3 and MCM4) were run on qRT-PCR for a subset of 42 cancer tissues and their adjacent non-neoplastic tissues. Compared with non-tumor larynx tissues, the mRNA expression levels of the 6 genes in LSCC tissues were statistically different (P <0.05), and the results of cancer tissues were significantly up-regulated. The result was shown below in Table 8.

**Table 8:** QRT-PCR analysis between LSCC tissues and their corresponding adjacent non-neoplastic tissues (paired sample t tests).

genes	relative mRNA expression levels		
	carcinoma tissues	non-neoplastic tissues	P
mcm2	1.57 ±1.02	1.05±0.62	0.037
mcm3	1.12±0.84	0.96±0.71	0.045
mcm4	1.61±0.92	1.08±0.54	0.012
CDK1	2.53±1.23	1.52±0.98	0.005
CDK2	2.78±1.65	1.15±0.74	0.005
CDK4	2.92±1.22	1.02±0.60	0.001

### Gene expression analysis of regional lymph node metastasis in LSCC

**Figure 3:**
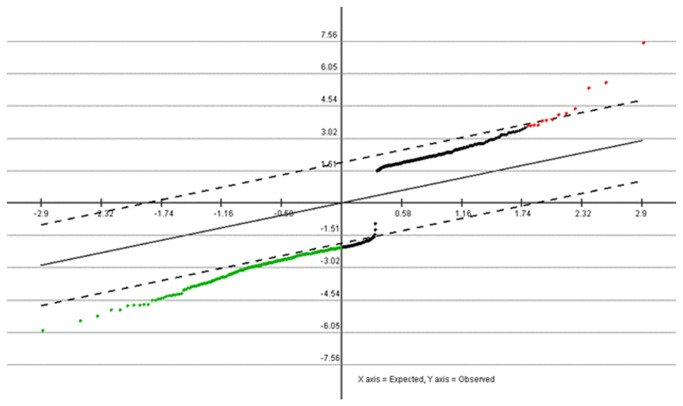
SAM Graph of genes related to regional lymph node metastasis in LSCC. Red points indicated higher expression genes (P<0.05) and green points indicated lower expression genes (P<0.05) across the 246 samples.

**Figure 4:**
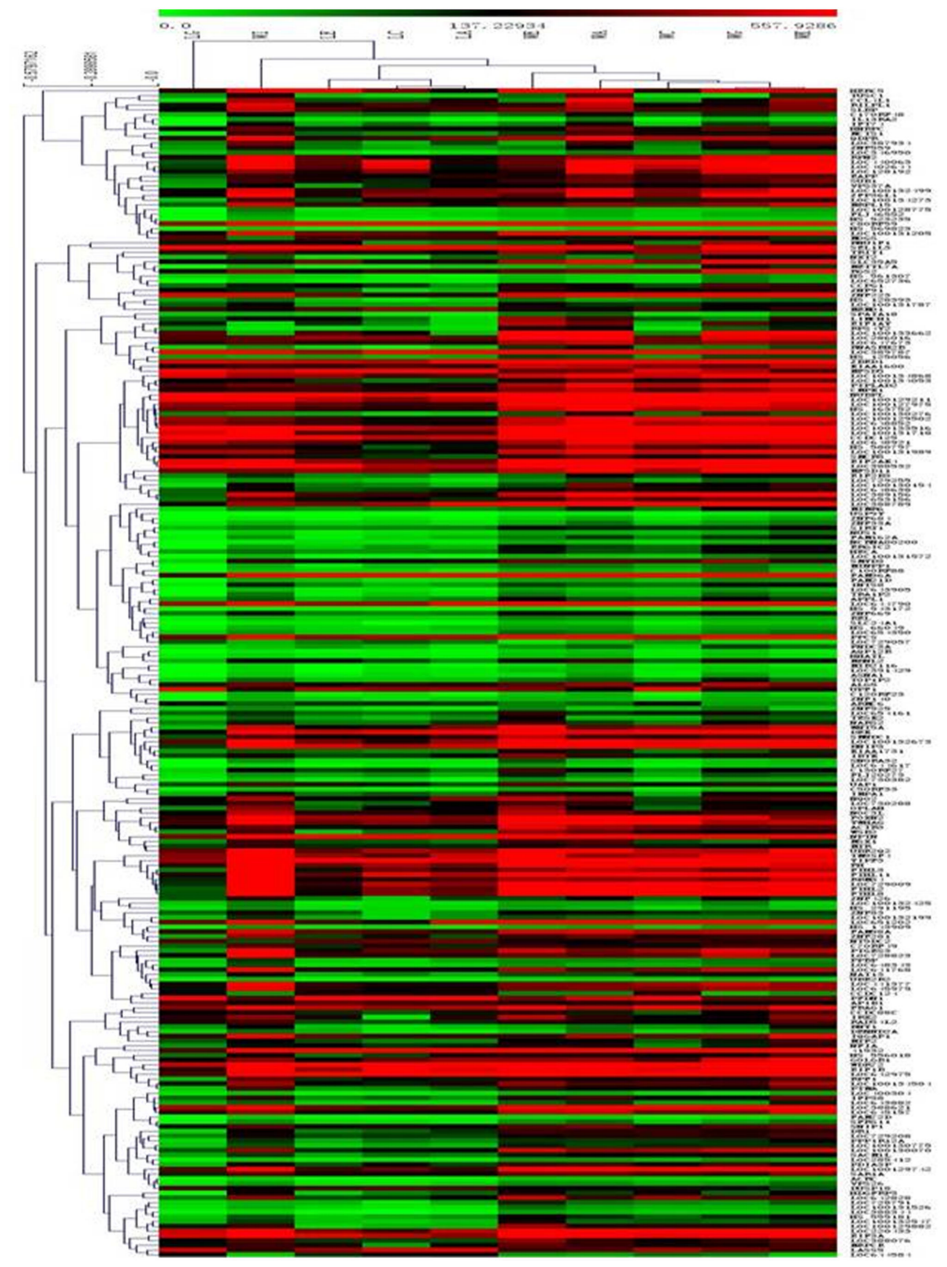
Hierarchical Trees:All significant genes of regional lymph node metastasis in LSCC. For each gene (row), red indicated a higher expression and green a lower one relative to the average level of expression of that gene across the 246 samples (columns). To identify typical nuclear genes which might be therapeutic markers from the identified 246 genes, the GO database and the KEGG pathways database were also used for biological process enrichment analysis. Analyzed by GO database, we observed that these genes were involved in processes such as cellular macromolecule metabolic process, translation, organic substance biosynthetic process, biosynthetic process, cellular metabolic process, RNA binding and so on (P <0.05). RNA transport pathway and N-Glycan biosynthesis pathway played especially important role in regional lymph node metastasis of LSCC analyzed by KEGG pathways database(P<0.05). Of the 246 genes, we found that the genes such as eIF3a, eIF2b3, RPN2, UPF1, ALG3 were statistically related with regional lymph node metastasis of LSCC by the analysis of GO database and KEGG database. Among the significantly related genes, eIF3a and RPN2 were extremely representative genes in the two functional pathways analyzed by KEGG, and the expression levels in regional lymph node metastasis tissues were significantly down-regulated by mRNA microarrays analysis (The results are shown in Table 9, Table 10 and Table 11). Subsequently, qRT-PCR was used for validation.

The LSCC tissues with regional lymph node metastasis(5 samples) and LSCC tissues without regional lymph node metastasis (5 samples) were compared, and the methods were introduced in the previous paragraph. In this study, 246 genes showed statistically significant differences in the expression with regional lymph node metastasis (P <0.05). Among these genes, 13 genes showed a higher expression in tumors with regional lymph node metastasis, while 233 presented the contrasting pattern. Two subgroups of tumor samples were distinguishable in the training set based on the expression profile of the different genes, which suggested regional lymph node metastasis was affected by gene regulation in LSCC. The result is shown below in Figure 3 and Figure 4.

**Table 9:** Microarray analysis of two genes between tissues with regional lymph node metastasis or not.

genes	fold change	P
eIF3a	0.28	0.028
RPN2	0.47	0.035

**Table 10:** Two genes in GO database ID.

GO database (ID)	genes	P
**GO:0044260**	eIF3a, RPN2	0.0003
**GO:0006412**	eIF3a, RPN2	0.0003
**GO:0044267**	eIF3a, RPN2	0.0020
**GO:1901576**	eIF3a, RPN2	0.0016
**GO:0009058**	eIF3a	0.0022
**GO:0044237**	eIF3a, RPN2	0.0017
**GO:0003743**	eIF3a	0.0242
**GO:0004576**	RPN2	0.0019

**Table 11:** Two genes in KEGG pathways database ID.

KEGG pathways database (ID)	genes	P
**RNA transport(03013)**	eIF3a	0.0021
**N-Glycan biosynthesis (00510)**	RPN2	0.0089

 EIF3a, the largest subunit of eIF3 complex(translation initiation factor), has been shown to play a role in regulating synthesis of proteins including a-tubulin, ribonucleotide reductase M2, and p27 as well as in cell proliferation, cell cycle control and cell differentiation [25,26]. RPN2, part of an N-oligosaccharyl transferase complex, affects cell apoptosis and cell growth [27]. EIF3a and RPN2 were run on qRT-PCR for a subset of the LSCC tissues with regional lymph node metastasis (19 samples) and those without regional lymph node metastasis (23 samples). Compared with non-regional lymph node metastasis cancer tissues, the mRNA expression levels of the 2 genes in regional lymph node metastasis cancer tissues were both statistically different (P <0.05),and the results in regional lymph node metastasis cancer tissues were significantly down-regulated. The results of the 2 genes are shown in Table 12. Because both eIF3a and RPN2 reacted to docetaxel [28,29], they might be useful target markers which should be payed sufficient attention.

**Table 12:** QRT-PCR analysis between tissues with regional lymph node metastasis or not (independent sample t tests).

Genes	relative mRNA expression levels		
	metastasis	no metastasis	P
eIF3a	0.85 ±0.52	1.17±0.74	0.038
RPN2	0.76±0.50	0.98±0.61	0.042

## Discussion

The mRNA microarray is proving to be a valuable resource for biomarker identification. In this study, we found a substantial number of new differentially expressed genes affecting tumorigenesis and regional lymph node metastasis in laryngeal squamous cell carcinoma at an FDR ≤ 0.05. Illumina Human HT-12 BeadChip was chosen, as it could detect the expression levels of 34601 genes in our clinical samples. This is the first research involving mRNA microarray analysis to determine gene expression changes during disease development and progression in LSCC. 

 We identified 361 genes as differentially regulated in LSCC tissues as compared to corresponding non-neoplastic tissues. Among these 361genes, 232 showed a higher expression in tumor than in non-tumor tissue, and 129 presented the contrasting pattern. The differentially expressed genes were mainly involved in processes such as mitosis, cell cycle phase, cell cycle process, ATP-banding, apoptosis, nuclear division and so on. At the molecular level, six genes (CDK1, CDK2, CDK4, MCM2, MCM3 and MCM4) were the most frequently selected genes affecting tumorigenesis in LSCC, and they were also validated by qRT-PCR. MCM2, MCM3 and MCM4 are minichromosome maintenance proteins, which are essential for DNA replication in all eukaryotic cells and for restricting replication to once per cell cycle [23]. Minichromosome maintenance protein (MCM) is a family of six highly conserved and highly homologous proteins (MCM2-7). The MCM2-7 polypeptides form a functional hexameric complex [30] that comprises an important part of the ‘prereplicative complex’ of replication proteins at replication origins during the G1 phase. The protein then irreversibly dissociates to ensure that DNA synthesis is initiated only once during each cell cycle [31] and not evident in quiescent, differentiated and senescent cells, and all of the six MCM proteins show similar and comparable expressions in a range of tissue sections [32]. In previous study, MCM2, MCM3 and MCM4 were dysregulated in malignant salivary gland tumours [33], gastric cardiac cancer [34], thyroid malignancy [35], non-small cell lung cancer [36], malignant melanoma [37], colon cancer, promyelocytic leukemia [38], cervical squamous cell carcinoma [39]. In our research, the high expression of the 3 genes consequently contributed to larynx carcinogenesis, which suggested they might be useful target markers.

 It is also known that cyclin-dependent kinases (CDKs) interact at specific stages of the cell cycle to drive the cell cycle from one phase to the next in cells. For example, CDK1/Cyclin B complex plays an important role for regulation of G2/M phase [40,41]. CDK2-cyclin E complex is known to initiate both DNA replication and centrosome duplication during the G1-S transition in the cell cycle [24]. Constitutive expression of CDK4 results in hyperphosphorylation of Rb and increased E2F activity, leading to inappropriate progression through the G1/S phase of the cell cycle [40]. In previous study, the genes (CDK1, CDK2 and CDK4) were dysregulated in breast cancer [42], ovarian cancer [43], colon cancer [44], hepatocellular carcinoma [45], thyroid carcinoma [46], and lung cancer [47]. We also found that the high expression of CDK1, CDK2 and CDK4, part of cyclin-dependent kinases, were related to tumorigenesis in LSCC, and the results were validated by qRT-PCR. They were also useful target markers related to tumorigenesis as the genes (MCM2, MCM3 and MCM4) mentioned previously in the study.

 Moreover, analyzed by drug association database, CDK1 was related to paclitaxel, mechlorethamine and CDK2 was related to mechlorethamine. This result indicated that CDK1 and CDK2 also might be therapeutic target genes.

 We also investigated the genes related to regional lymph node metastasis besides tumorigenesis. Regional lymph node metastasis plays an important role as a prognostic factor in laryngeal squamous cell carcinoma. Research has been carried out for many years to pinpoint the factors, which facilitate spreading of the tumor into lymph nodes. However, it is still difficult to give explicit results [48]. This study used by microarrays analysis revealed that some functional molecules were crucial for malignant cells to metastasize in molecular biology. The LSCC tissues with regional lymph node metastasis those without regional lymph node metastasis were compared, and 246 genes were identified as differentially regulated. Among these genes, 13 genes showed a higher expression in tumors with regional lymph node metastasis, while 233 presented the contrasting pattern. Being different from the genes related to tumorigenesis, these genes were mainly involved in processes such as cellular macromolecule metabolic process, translation, organic substance biosynthetic process, biosynthetic process, cellular metabolic process, RNA binding and so on. The result indicated that the basis of molecular biology was different between tumorigenesis and regional lymph node metastasis in laryngeal squamous cell carcinoma, which suggested that disease development and progression of LSCC were differently progressive processes. Analysed by GO database and KEGG pathways database, eIF3a and RPN2 which were low-expression in regional lymph node metastasis tissues were the most frequently selected genes affecting regional lymph node metastasis in our study and validated by qRT-PCR. As the function of eIF3a and RPN2 had been previously discussed, they need to draw sufficient attention.

 EIF3a appeared to be essential for cancer cells to maintain malignant phenotype. Suppressing endogenous eIF3a expression had been shown to reverse the malignant phenotype of human cancer cells and overexpression of eIF3a had been found in many cancers such as cancers of lung, breast, cervix, stomach, and esophagus [49]. However, it has been observed previously that cervical and esophageal cancer patients with high eIF3a level had better relapse-free and overall survival than those with low eIF3a expression [50,51]. Moreover, when human lung cancer A549 cells were treated with high concentration of docetaxel, the expression level of eIF3a mRNA tended to increase in a time-depend manner. Docetaxe could slightly increase the expression level of eIF3a mRNA [28]. EIF3a upregulation in lung cancer patients also correlated with their response to platinum-based chemotherapy and contributed to increased cisplatin (cis-dichlorodiammine platinum(II) (CDDP)) sensitivity [52]. These observations suggest that eIF3a has an important role in cancer cell response to chemotherapeutics, possibly by regulating gene expression.RPN2 may also have an significant role in cancer cell response to chemotherapeutics. Recently, Honma et al [27] reported that downregulation of ribophorin II (RPN2)) promoted docetaxel-dependent apoptosis and cell growth inhibition of MCF7-ADR human breast cancer cells that are resistant to docetaxel It also has been found that RPN2 suppression increased sensitivity to docetaxel in oesophageal squamous cell carcinoma in vitro [53]. Considering our detection, expression of RPN2 in biopsy specimens could be a useful predictive marker for response to neoadjuvant chemotherapy in LSCC.

## Conclusion

In conclusion, our microarray analysis revealed a gene expression signature of tumorigenesis and regional lymph node metastasis in laryngeal squamous cell carcinoma, and several genes whose deregulation is potentially associated with disease development and progression were validated by qRT-PCR. Further studies of the genes are required to explore the specific mechanisms and evaluate the clinical application values. Our findings will contribute to the understanding of the molecular basis of laryngeal squamous cell carcinoma, thus helping to improve diagnosis and treatment. 
